# Freedom of speech for all critically ill patients: work in progress

**DOI:** 10.1186/s13054-017-1608-2

**Published:** 2017-02-08

**Authors:** P. R. Tuinman, S. ten Hoorn

**Affiliations:** 1 Department of Intensive Care Medicine and Research VUmc Intensive Care (REVIVE), VU University Medical Center Amsterdam, Room ZH—7D-166, De Boelelaan 1117, PO Box 7057, MB Amsterdam, 1007 The Netherlands; 2 Institute for Cardiovascular Research VU (ICaR-VU), VU University Medical Center Amsterdam, Amsterdam, The Netherlands

Freedom of speech, e.g. to seek, receive and impart information, is one of the most precious rights of man. Both Egbers and Boerma [1] and Sutt and Fraser [2] highlight the possibility of using an (in-line) speaking valve in ventilator-dependent patients to improve communication through speech and suggest adding this option to the algorithm presented in our systematic review on communication with these patients [3]. As already pointed out by the authors, this option was beyond the scope of our literature search. However, we thank the authors for their suggestions, because using the patient’s own voice is a very important option for improving communication with mechanically ventilated patients, and underlines three important issues regarding updating the algorithm for selecting alternative communication methods.

First, the scope of the algorithm may be broadened to critically ill patients who can tolerate cuff deflation and/or patients with communication problems due to other reasons, such as non-invasive ventilation or intensive care unit-acquired weakness.

Second, new innovative communication techniques will be introduced in the future and need to be added to the algorithm. Next to the inclusion of speaking valves for restoration of the patient’s own voice [4] as suggested by Sutt, Egbers and their co-workers, we think that developments in mobile technology will be of special interest. For example, mobile communication apps for tablets may benefit patients and replace simple communication boards in the future. These apps have not so far been studied and therefore have not yet been added to the algorithm. Also, we recently studied a new promising speech enhancement device. This device consists of a sensor which is placed on the patient’s neck to conduct the faint vocalizations of the patient to a control unit, where the signal is amplified to produce an audible sound [5]. Especially, patients with non-invasive ventilation, a weak voice or patients with a tracheotomy seem to benefit.

Third, next to updating the algorithm with new communication methods, it also needs to be adjusted to the experience, preferences and resources of individual intensive care units. Therefore, depending on the availability of high-tech augmentative communication devices and the training, experience and preferences for the different alternative communication methods of the intensive care unit staff, a local algorithm should be designed.

In this way, each individual intensive care unit has its own tailor-made algorithm to maximize effective communication with critically ill patients, thereby allowing patients to seek, receive and impart information.
